# Immunotherapy of HBV-related advanced hepatocellular carcinoma with short-term HBV-specific TCR expressed T cells: results of dose escalation, phase I trial

**DOI:** 10.1007/s12072-021-10250-2

**Published:** 2021-11-30

**Authors:** Fanping Meng, Jinfang Zhao, Anthony Tanoto Tan, Wei Hu, Si-Yu Wang, Jiehua Jin, Juan Wu, Yuanyuan Li, Lei Shi, Jun-Liang Fu, Shuangjie Yu, Yingjuan Shen, Limin Liu, Junqing Luan, Ming Shi, Yunbo Xie, Chun-Bao Zhou, Regina Wanju Wong, Wai Lu-En, Sarene Koh, Antonio Bertoletti, Tingting Wang, Ji-Yuan Zhang, Fu-Sheng Wang

**Affiliations:** 1 Senior Department of Infectious Diseases, The Fifth Medical Center of Chinese PLA General Hospital, National Clinical Research Center for Infectious Diseases, 100 Western 4th Ring Road, Beijing, 100039 China; 2 Emerging Infectious Diseases, Duke-NUS Medical School, Singapore, Singapore; 3 Lion TCR Pte. Ltd, 77 Ayer Rajah Crescent, #01-29, Singapore, 139954 Singapore; 4 Singapore Immunology Network, Agency for Science and Technology (A*STAR), Singapore, Singapore

**Keywords:** HBV, HBV-TCR-T cells, HCC, Safety, Chronic hepatitis B, Clinical trial, Phase 1, Immunotherapy, Overall survival, Time-to-progression

## Abstract

**Background & aims:**

Immunotherapy with hepatitis B virus (HBV)-specific TCR redirected T (HBV-TCR-T) cells in HBV-related hepatocellular carcinoma (HBV-HCC) patients after liver transplantation was reported to be safe and had potential therapeutic efficacy. We aim to investigate the safety of HBV-TCR-T-cell immunotherapy in advanced HBV-HCC patients who had not met the criteria for liver transplantation.

**Methods:**

We enrolled eight patients with advanced HBV-HCC and adoptively transferred short-lived autologous T cells expressing HBV-specific TCR to perform an open-label, phase 1 dose-escalation study (NCT03899415). The primary endpoint was to evaluate the safety of HBV-TCR-T-cell therapy according to National Cancer Institute Common Terminology Criteria for Adverse Events (version 4.03) during the dose-escalation process. The secondary endpoint was to assess the efficacy of HBV-TCR-T-cell therapy by evaluating the anti-tumor responses using RECIST criteria (version 1.1) and the overall survival.

**Results:**

Adverse events were observed in two participants among the 8 patients enrolled. Only one patient experienced a Grade 3 liver-related adverse event after receiving a dose of 1 × 10^5^ HBV-TCR-T cells/kg, then normalized without interventions with immunosuppressive agents. Among the patients, one achieved a partial response lasting for 27.7 months. Importantly, most of the patients exhibited a reduction or stabilization of circulating HBsAg and HBV DNA levels after HBV-TCR-T-cell infusion, indicating the on-target effects.

**Conclusions:**

The adoptive transfer of HBV-TCR-T cells into advanced HBV-HCC patients were generally safe and well-tolerated. Observations of clinical efficacy support the continued development and eventual application of this treatment strategy in patients with advanced HBV-related HCC.

**Clinical trials registration:**

This study was registered at ClinicalTrials.gov (NCT03899415).

## Introduction

Hepatocellular carcinoma (HCC) is one of the most common cancers worldwide, and is characterized by rapid progression and poor prognosis with 5-year survival rates of less than 5% [1, 2]. Surgical resection, chemotherapy and radiotherapy are conventional treatments on HCC, but, in the end, most patients die from disease recurrence or metastasis. Tyrosine kinase inhibitors, such as sorafenib, lenvatinib and cabozantinib, had been approved for HCC, but poor improvement in overall survival of advanced HCC patients was observed in the clinic [3–5]. Immune checkpoint blockade (ICB) therapy is a newly developed treatment for HCC. The efficacy of ICB therapy alone or in combination with kinase inhibitors has been reported with varying response rates and survival benefits in HCC patients [6–13]. Despite progress in available therapies, effective and durable systemic treatment options for HCC are still limited.

Adoptive immune cell therapies against HCC have been used by administrating living immune cells with or without antigen specificity. For example, non-specific adoptive cell therapy, including lymphokine-induced killer cells, autologous cytokine-induced killer (CIK) cells and natural killer cells, has captured increasing attention [14–16]. These immune-cell-based therapies, in combination with conventional treatments, and/or kinase inhibitors, demonstrate good safety in HCC patients. However, the lack of specificity restricts these immunotherapies primarily as adjuvant treatments. Specific adoptive cell therapy, such as chimeric antigen receptor engineered T-cell and T cell receptor-engineered T-cell (TCR-T) therapy, was hence developed to enhance the anti-tumor effect and improve treatment efficacy [17].

Hepatitis B-related HCC (HBV-HCC) is estimated to account for approximately 75–80% HCC cases in China [18, 19]. Furthermore, more than 90% of HBV-HCC have HBV DNA integrations and may express the whole or truncated forms of HBV antigens [20–22], so it is reasonable to presume that HBV T cell epitopes are processed and presented on HBV infected hepatocytes or HCC cells. Thus, it seems feasible to treat HBV-HCC using T cells engineered with HBV-specific T cell receptors (HBV-TCR T cells). Indeed, immunotherapy with HBsAg-specific TCR redirected T cells in three HBV-HCC patients after liver transplantation was reported to be safe and had potential therapeutic efficacy [22, 23]. However, at the moment there are no evidences to support whether HBV-TCR T cell could be implemented safely and with sufficient efficacy in advanced HBV-HCC patients due to the increased risk of severe liver inflammation from the destruction of HBV-infected normal hepatocytes (on-target off-tumor effects) under the general poor physical condition of these patients.

In the current phase I clinical trial, we examined the safety of T cell therapy using short-lived HBV-TCR-T cells in eight patients with advanced HBV-HCC. Our study supports the notion that HBV-TCR-T cell therapy is safe and well tolerated. On this basis, we also preliminarily evaluated the potential clinical efficacy of the treatment.

## Patients and methods

### Workflow and clinical design

Here, we adoptively transferred autologous short-lived mRNA HBV-TCR-T cells to perform an open-label, phase 1 dose-escalation study (NCT03899415) in eight advanced HBV-HCC patients from the Fifth Medical Center of Chinese PLA General Hospital, Beijing, China. This study was approved by the Ethics Committee of the Fifth Medical Center of PLA General Hospital and was conducted according to the principles of the Declaration of Helsinki.

**Fig. 1:**
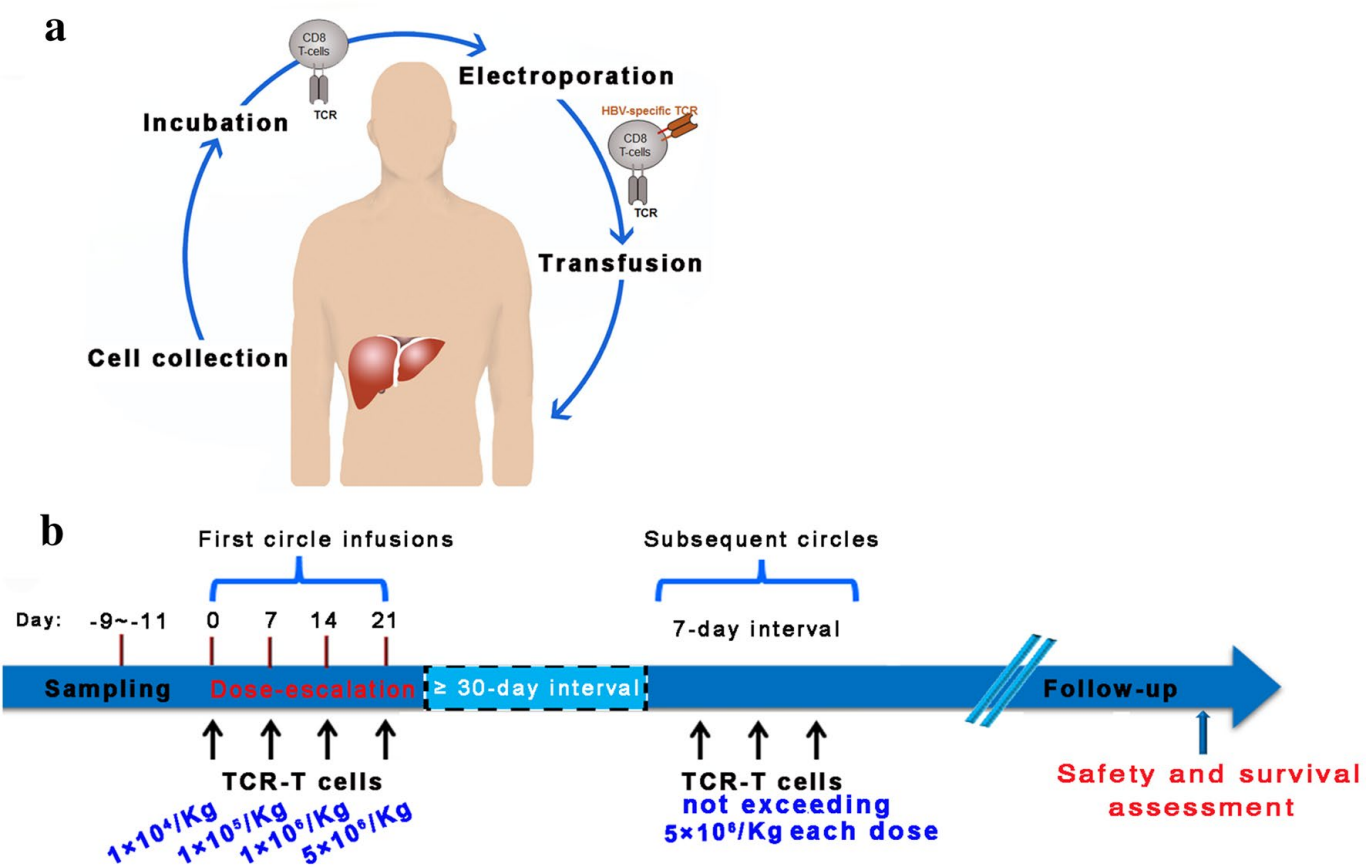
Workflow and schematic of study design. **a** The Workflow of HBV-TCR-T-cell therapy. PBMCs are harvested, expanded, redirected short-lived mRNA HBV-Env-specific-TCR by electroporation and transferred back into the same patient. **b** The clinical protocol showing the overall study design included the infusion cycle, infusion dose and the main outcomes

The workflow was showed in Fig. 1a. In this study, patient B004 was HLA-Cw0801 type and the remaining patients (B001-B003 and B005-B008) were HLA-A0201 type (Table 1). TCR-A2-HBs183-191 (TCR-A02/HBs) was used for the HLA-A0201 patients, and TCR-HBV s171 (TCR-C08/HBs) was used for HLA-Cw0801 patients (Table 1). The production of HBV-TCR-T cells were performed as follows: peripheral blood mononuclear cells (PBMCs) were harvested from 80 mL anti-coagulated whole blood of enrolled subjects, followed by the activation and expansion for 8 days with IL-2 (600 IU/mL, Miltenyi) and OKT-3 (50 ng/mL, Miltenyi) in AIM-V (Invitrogen) with 5% CTS Serum Replacement (Invitrogen) as demonstrated in the previous study [15]. Then, activated T cells were manufactured to express HBV-specific TCR through electroporation [22], and cultured overnight in AIM-V with IL-2 (100 IU/mL, Miltenyi). HBV-TCR-T cells were collected by centrifugation for 20 min at 1500 r/min, washed twice in saline (containing 5 g/L human albumin), resuspended in the same solution with 400–500 mL, then transfused back into patients intravenously.

**Table 1:** Characteristics of patients

Patients No	B001	B002	B003	B004	B005	B006	B007	B008
Gender	Male	Male	Male	Male	Male	Male	Male	Male
Age (years)	49	51	59	48	46	67	56	59
AST (IU/L) at baseline	28	44	71	71	26	42	80	19
ALT (IU/L) at baseline	22	36.5	40	48	26	27.5	28	13
Total bilirubin (μmol/L) at baseline	8.5	43.3	18	21.7	7.5	11.1	15.3	10.1
Platelet counts (10^9^/L) at baseline	230	24	77	173	160	192	276	147
HBV‐TCRs (HBV‐Env)	HLA‐A0201	HLA‐A0201	HLA‐A0201	HLA‐Cw0801	HLA‐A0201	HLA‐A0201	HLA‐A0201	HLA‐A0201
HBV genotype	C	–	C	C	–	–	–	
HBsAg serum (IU/ml)	202	1444	929.9	210.9	942.6	1549	231.5	303.8
HBeAg status	Negative	Negative	Negative	Negative	Negative	Negative	Negative	Negative
HBeAb status	Positive	Positive	Positive	Positive	Positive	Positive	Positive	Positive
Circulating HBV DNA (IU/ml) at baseline	499	< 40	458	991	< 20	44.2	< 20	< 20
Epitope sequence at baseline	FLLTRILTI	–	FLLTRILTI	FLGPLLVLQA	–	–	–	–
Circulating HBV DNA (IU/ml) after TCR-T-cell infusions	30.5	< 40	318	492	< 40	< 20	< 20	< 20
Epitope sequence after TCR-T-cell infusions	–	–	FLLTRILTI	FLGPLLVLQA	–	–	–	–
AFP ≥ 400 ng per milliliter	No	No	No	Yes	No	No	Yes	No
ECOG performance status score	1	1	2	2	0	1	1	1
Child–Pugh classification (score)	A5	B8	A6	B9	A5	A6	A6	A6
BCLC stage	C	B	C	C	C	A	C	C
Tumor in liver (size, max, cm & number)	Multiple (13.9 × 6.9)	Multiple (3.5 × 1.9)	Multiple (4.8 × 13.1)	Multiple (17.9 × 12.1)	2 (3.9 × 3.4)	1 (3.2 × 3.07)	Multiple (11.5 × 9.3)	2 (3.8 × 3.2)
Presence of Macrovascular invasion	Yes	No	Yes	Yes	No	No	No	No
Presence of Extrahepatic spread	Yes	No	Yes	Yes	Yes	No	Yes	Yes
Antiviral treatment	Entecavir	Entecavir	Entecavir	Entecavir	Entecavir	Entecavir	Entecavir	Entecavir
Prior treatment for HCC	TACE + Sorafenib	TACE + Sorafenib	RFA	Sorafenib	TACE + Sorafenib	TACE + RFA	Sorafenib	Argon-helium knife therapy for HCC + RFA + Sorafenib
Combination therapy	Sorafenib	Sorafenib	Sorafenib	Sorafenib	Sorafenib + 1 RFA	1 Microwave ablation	Sorafenib	Sorafenib
Therapy after TCR-T-cell infusion	Sorafenib	Sorafenib + Lenvatinib + 2 TACE	Sorafenib	Sorafenib	Sorafenib + 1 RFA	no	Sorafenib	Sorafenib

*AST* aspartate aminotransferase, *ALT* alkaline phosphatase; *HBV* hepatitis B virus, *HBsAg* hepatitis B surface antigen, *HBeAg* hepatitis B e-antigen, *AFP* α-fetoprotein; *ECOG* Eastern Cooperative Oncology Group, *BCLC* Barcelona clinic liver cancer (BCLC) stage, *TACE* transcatheter arterial chemoembolization; *RFA* radiofrequency ablation

The patients were admitted to the hospital before cell therapy. No lymphodepletion was performed prior to the adoptive transfer of HBV-TCR-T cells. The treatment schedule with HBV-TCR-T cells was shown in Fig. 1b: the first cycle included 4 escalating doses (1 × 10^4^–5 × 10^6^ CD8^+^Vβ^+^ T-cells/kg) at one-week interval, and the subsequent infusions were given with variable quantities of HBV-TCR-T cells at a maximum dose of 5 × 10^6^ CD8^+^Vβ^+^ T-cells /kg, each given one-week apart.

The primary endpoint was to evaluate the safety of HBV-TCR-T-cell therapy, and the dose-limiting toxicity of TCR-T cells was defined as follows: Grade 3/4 adverse events not related to patient’s underlying malignancy or preexisting comorbidities or any unexpected toxicity of any grade according to National Cancer Institute Common Terminology Criteria for Adverse Events (version 4.03) during dose-escalation process and follow-up period. The secondary endpoint was to assess the efficacy of HBV-TCR-T-cell therapy by measuring tumor response as follows: the first evaluation of the tumor response was performed at the 21st to 24th post days from the first TCR-T-cell infusion by MRI or CT according to RECIST criteria (version 1.1), and every 3 months thereafter. Overall survival (OS) was assessed and analyzed [24].

### Enrollment criteria of patients

Eligible patients were (1) aged 18–70 years; (2) expressing either HLA-A0201 or HLA-Cw0801; (3) Barcelona clinic liver cancer (BCLC) stage A-C HCC patients with a positive test for HBsAg; (4) at least 1 month after a surgical intervention or 2 weeks after transhepatic arterial chemotherapy and embolization (TACE) (may include other type of resection/ablation); (5) Child–Pugh < 7 points and Eastern Cooperative Oncology Group (ECOG) ≤ 1; (6) alanine aminotransferase (ALT) < 200 IU/L, aspartate aminotransferase (AST) < 200 IU/L, total bilirubin < 17.1 umol/L and creatinine clearance ≥ 60 ml/minute; (7) without clinically significant abnormality in chest X-ray, cardiac enzymes and electrocardiograph; (8) received antiviral treatment > 1 year prior to enrollment; (9) willing to use an acceptable method of contraception who was during child-bearing period; and (10) provided written informed consent.

Exclusion criteria were as follows: (1) patients experiencing acute infection or gastric bleeding within 30 days; (2) with positive hepatitis A/C/delta virus, human immunodeficiency virus, or a chronic liver disease other than CHB (e.g. autoimmune hepatitis, alcoholic liver disease, non-alcoholic fatty liver disease and drug-induced liver disease; (3) with any other serious physical and mental illnesses; (4) women who are pregnant or breast-feeding; (5) with a history of allergic reaction to blood products or other investigational products; (6) patients s who are receiving systemic medications, such as steroids during the study treatment; (7) any cell therapy such as, but not limited to natural killer, cytokine-induced killer, dendritic cells, cytotoxic T lymphocyte, stem cells therapy 6 months prior to study treatment.

### Detection of engineered HBV-TCR-T cells

To detect the frequencies of HBV-TCR-T cells, the following antibodies were used: anti-CD3-APC-Cy7, anti-CD8-BV510, and anti-TCR-Vβ. Cells were labelled with above-mentioned antibodies on ice for 30 min and then thoroughly washed and fixed for further analysis by flow cytometry.

### Quantification of circulating HBsAg and HBV DNA

HBsAg was quantified using a Roche Cobas e601 electrochemistry luminescence immunity analyzer and an elecsys for HBsAg quantitation (Roche Diagnostics, Mannheim, Germany) with a lower limit of detection: 0.05 IU/mL [25]. HBV DNA was measured from 200-μl plasma using HBV-DNA assay kit (SANSURE BIOTECH INC., Hunan, China) with a lower limit of detection: 40 IU/mL, or COBAS AmpliPrep/COBAS TaqMan (Roche Diagnostics, Mannheim, Germany) with a lower limit of detection: 20 IU/mL.

### HBV genotype and epitope identification

HBV genotype was determined as in previous study [26], and HBV DNA was sequenced to analyze HBV epitope sequences according to previous protocols [27].

### Statistical analysis

AE terms were coded using the Medical Dictionary for Regulatory Activities (version 21.1). Time-to-Progression (TTP) was analyzed from the date of HBV-TCR-T-cell infusion to the date of disease progression. TTP and OS were analyzed with the Kaplan–Meier method.

## Results

### Patient characteristics

The enrolled patients with advanced HCC were all male and had a median age of 53.5 (ranging from 46 to 67) years. Baseline characteristics of the patients were listed in Table 1. Liver function of all patients met the enrollment criteria prior to treatment. Six patients had Child–Pugh class A liver function (B001, B003, B005-B008) and two patients were of Child–Pugh class B (B002 and B004). As for BCLC staging, 6 patients had stage C (B001, B003-B005, B007 and B008); two patients had stage B (B002) and stage A (B006) HCC, respectively. B006 had a single liver lesion while the remaining patients had more than 1 hepatic tumors. In addition, three patients had macrovascular invasion and six patients had extrahepatic disease (Table 1). Two subjects (B004 and B007) had AFP ≥ 400 ng/mL, the ranges of serum HBsAg were from 202 IU/mL to 1549 IU/ml, and four patients (B001, B003, B004 and B006) had circulating HBV DNA level > 40 IU/mL (Table 1). Meanwhile, HBV genotypes in three patients (B001, B003 and B004) were C, and the corresponding HBV Epitope sequences of B001 and B003 were FLLTRILTI and B004 was FLGPLLVLQA (Table 1). The results suggest that the amino acid sequences recognized by TCR-T were conserved in B003 and B004 with the same sequence at baseline and after TCR-T-cell infusions (Table 1) Due to the low levels of circulating HBV DNA in the remaining six patients, we failed to test HBV genotypes in these patients. Other clinical parameters of enrolled patients including peripheral platelet counts, serum HBeAg and HBeAb status were also shown in Table 1.

All patients had a history of chronic HBV infection and received entecavir treatment. Apart from B003 and B006, other patients were administrated with sorafenib before TCR-T-cell therapy. Moreover, all other patients, with the exception of B004 and B007, received local therapy for liver tumor including TACE or liver lesion microwave ablation before TCR-T-cell therapy. Patients B001, B002, B003, B004, B005, B007 and B008 received Sorafenib treatment in combination with TCR-T cell infusion (Table 1). More detailed, B002 received Sorafenib first, then followed by Lenvatinib after 1 year of the last infusion, and underwent twice TACE after 1 and 2 year of the last infusion during the follow-up period; B005 received RFA at 1 month after the last infusion; and B006 underwent 1 microwave ablation after the first TCR-T-cell infusion (Table 1).

### Short-term HBV-TCR expression on T cells

**Fig. 2:**
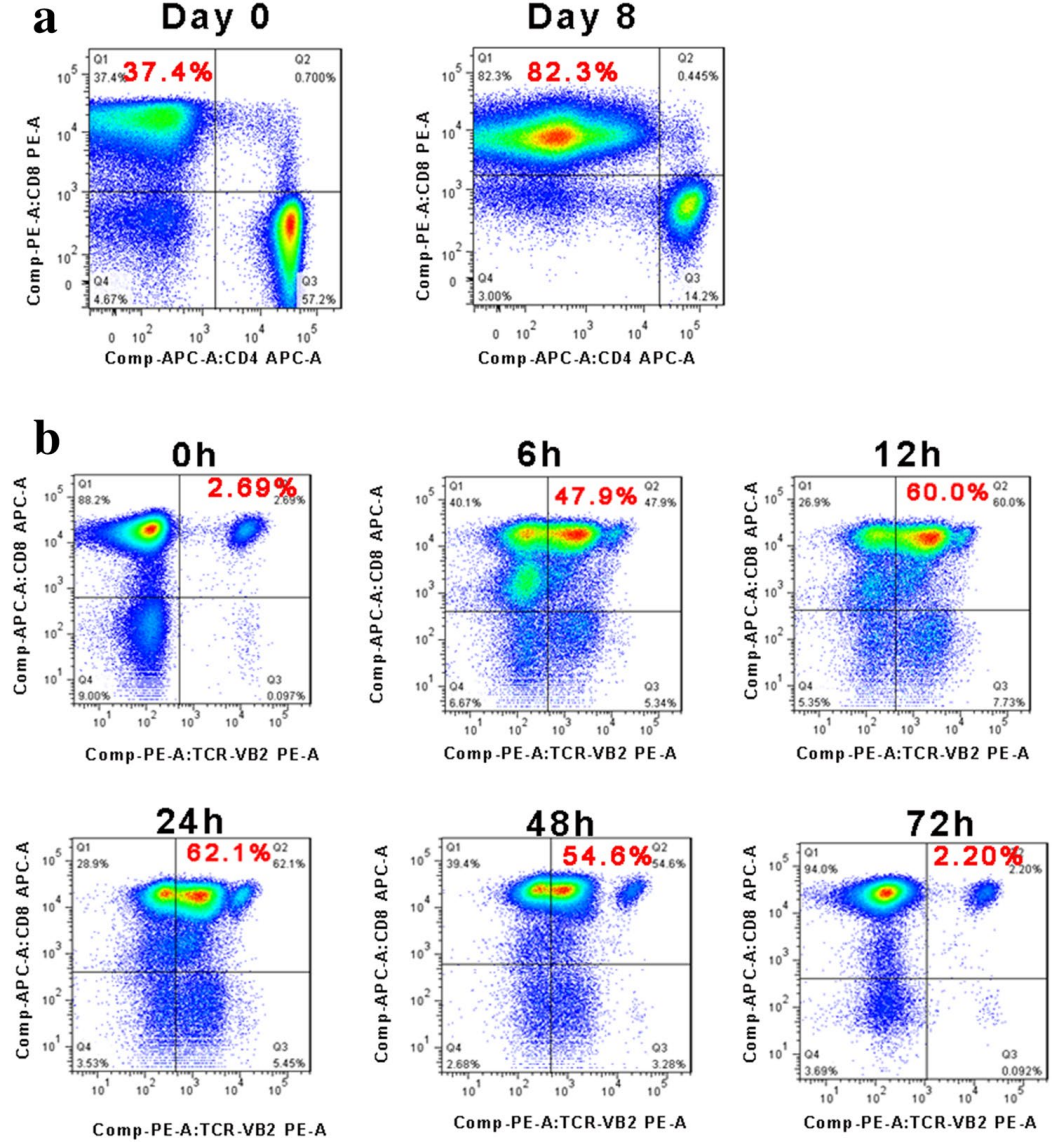
Characteristics of HBsAg-TCR-T cells. **a** Proliferation profiles of CD8 T cells from one HBV-HCC patient. Values in quadrant indicate percentage of CD8 T cells in cultured lymphocytes (gated on CD3 + T lymphocytes). **b** Electroporation efficiency. Values in quadrant indicate TCR-Vβ percentages expressed on T cells at different times after electroporation

We successfully generated HBV-TCR-T cells for all eight patients. The median time to manufacture the cell products for clinical use was 10 (range from 9 to 11) days. Before electroporation, CD8 T cells accounted for 85% (range from 79 to 93%) of expanded cells after in vitro expansion. Representative dot plots of the percentages of CD8^+^ T cells in CD3^+^ T cells from B001 were shown in Fig. 2a. The percentages of HBV-TCR-T (CD8^+^Vβ^+^) cells in CD3^+^ T cells remained high between 12 and 48 h post electroporation, and then decreased to normal levels after 72 h. Representative dot plots of the dynamic percentages of HBV-TCR-T (CD8^+^Vβ^+^) cells in CD3^+^ T cells from B001 were shown in Fig. 2b. Given the clinical application, we performed infusions with HBV-TCR-T cells obtained 24 h after electroporation.

### Adverse effects

**Fig. 3:**
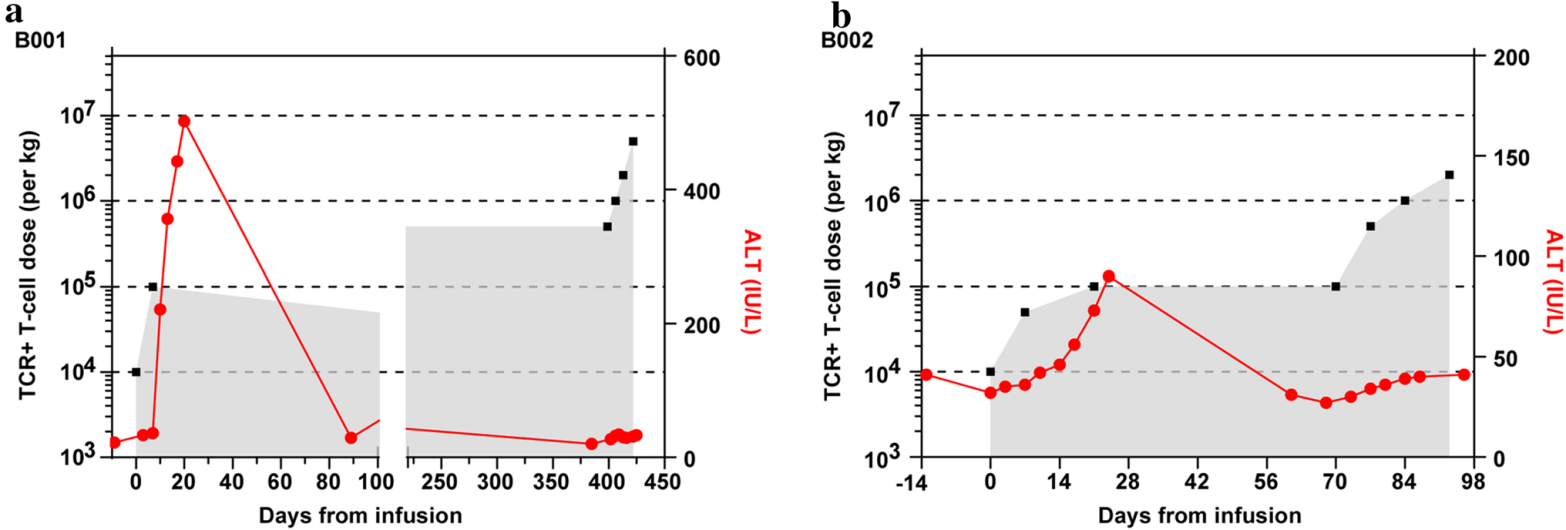
The alterations of ALT levels in B001 and B002 after TCR-T-cell infusion **a** B001 and **b** B002

The median numbers of cell infusions were 8 (range from 4 to 12), B001 received 12 cell infusions, B002, B007 and B008 8 infusions, and B003-B006 received 4 infusions, respectively. During and immediately after T cell infusions, none of patients had acute adverse events of vomiting, allergies, coma, or graft-versus-host disease. The incidence and severity of all observed toxicities and their associated dose are displayed in Table 2. Two patients (B001 and B002) experienced adverse events during the first cycle of HBV-TCR-T infusion. B001 experienced Grade 3 adverse events with a rapid elevation of liver enzymes (a max ALT of 506 IU/ml) and jaundice (a max total bilirubin of 70.3 µmol/L) after receiving the second dose (1 × 10^5^ cells/kg). These indexes peaked at ~ 16 days and resolved at ~ 80 days after the second TCR-T infusion (Fig. 3a). B002 had a mild and gradual elevation of ALT with a maximum of 90 IU/ml at ~ 10 days and resolved at ~ 50 days after the second infusion (5 × 10^4^ cells/kg) (Fig. 3b). In both patients, liver enzymes normalized without interventions with glucocorticoid or other immunosuppressive agents such as IL-6 receptor antagonist tocilizumab. No liver-related adverse events were observed during the subsequent second cycle HBV-TCR-T infusions. Adverse events were not observed for the rest of the patients even at the maximum planned dose of 5 × 10^6^ cells/kg. There were also no abnormal changes in renal function among the eight patients during the whole treatment period. Taken together, these data indicate that HBV-TCR-T-cell therapy for advanced HBV-HCC patients was safe and well tolerated.

**Table 2:** Safety evaluation

Patients No	The number of infusions and maximum dose	HBV TCR-T cell related AE		Best overall response	TTP (months)	OS (months)	Current status
		AE	Associated dose				
B001	12 (5 × 10^6^/kg)	Grade 3 ALT increase; Grade 3 GGT increase; Grade 3 AST increase; Grade 3 bilirubin increase	1 × 10^5^/kg	PR	27.7	37.4 (censored)	Loss to follow-up
B002	8 (2 × 10^6^/kg)	Grade 1 ALT increase	5 × 10^4^/kg	SD	2.97	33.1	Death
B003	4 (1 × 10^6^/kg)	None	NA	PD	0.93	3.53	Death
B004	4 (2 × 10^5^/kg)	None	NA	SD	0.9	2.53	Death
B005	4 (5 × 10^6^/kg)	None	NA	SD	9.4	18.8 (censored)	Loss to follow-up
B006	4 (1.53 × 10^6^/kg)	None	NA	NE	23.8	23.8 (censored)	Alive
B007	8 (5 × 10^6^/kg)	None	NA	PD	0.2	6.7	Death
B008	8 (5 × 10^6^/kg)	None	NA	NE	16.9	20.0 (censored)	Alive

*HBV* hepatitis B virus, *AE* adverse reaction, *TTP* time-to-progression, *OS* overall survival, *ALT* alanine transaminase, *GGT* gamma-glutamyltransferase, *AST* aspartate aminotransferase, *PR* partial response, *SD* stable disease, *PD* progressive disease, *NE* not evaluated, *NA* not available

### Antitumor activity of HBV-TCR-T-cell therapy

**Fig. 4:**
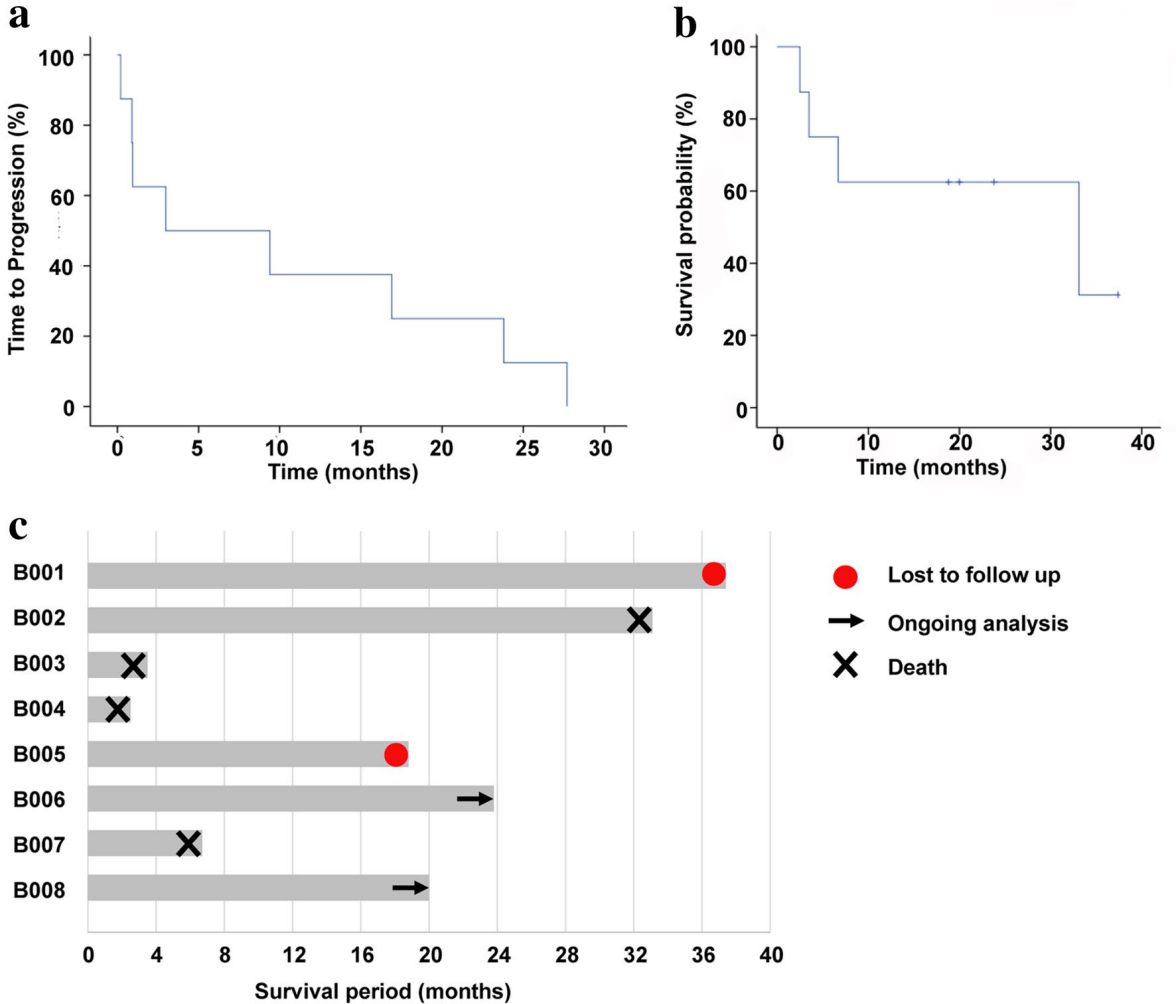
Disease response. **a** Median TTP for patients after infusions was 6.18 months *n* = 8, and **b** median OS for patients after infusions was 33.1 months (*n* = 8). **c** Outcome for evaluable patients by Swimmer plot

The impact on overall survival was then evaluated. The median TTP for patients enrolled was 6.18 months (ranging from 0.2 to 27.7 months) (Fig. 4a) and the median OS was 33.1 months (ranging from 2.5–37.4 months) (Fig. 4b). B003, B004 and B007 patients exhibited the shortest TTP (0.93, 0.9 and 0.2 months, respectively). The high tumor load and AFP values at baseline of these 3 patients together with extrahepatic and macrovascular spread at the inception of the study indicates a more advanced disease state before treatment (Table 1). B003 and B004 passed away within 4 months after one cycle of TCR-T-cell infusion and B007 died within 7 months after two cycles of TCR-T-cell infusion (Fig. 4c). On the other hand, B005, B006 and B008, showed a longer TTP (9.4, 23.8 and 16.9 months, respectively) and survival (18.8, 23.8 and 20.0 months, respectively) (Table. 2 and Fig. 4b). While B001 was lost to follow-up, B006 and B008 were alive at the time the data were analyzed (Table. 2 and Fig. 3c). The good outcomes of the three patients could be associated with the lower tumor load at study inception (Table. 1). This was especially pertinent for patient B006 who not only had a lower tumor load but was also at an earlier stage of the disease (BCLC stage A). Despite the high tumor load at baseline, B001 and B002 had a longer TTP (27.7 and 2.97 months, respectively) and survival (37.4 and 33.1 months, respectively) than those of B003, B004 and B007 after receiving 4 and 2 cycles of TCR-T-cell therapy, respectively (Fig. 4c). Importantly, B001 achieved a partial response lasting with 27.7 months, with a significant shrinkage of liver tumor size, and B002 achieved stable disease, supporting a significant benefit of HBV-TCR T-cell therapy on long-term OS. Overall, there was a trend towards increased survival after TCR-T treatment.

**Fig. 5:**
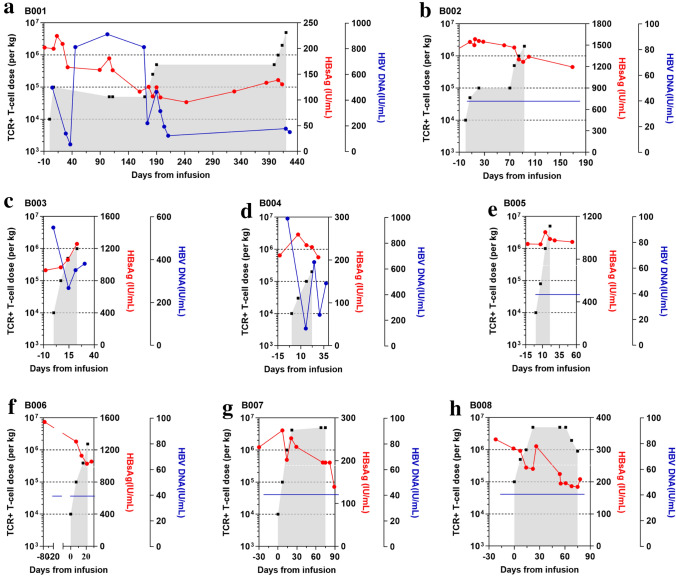
Serum levels of HBsAg and HBV DNA before and after TCR-T-cells infusion. HBsAg and HBV DNA levels of every patient treated with HBV-TCR-T cells **a** B001; **b** B002; **c** B003; **d** B004; **e** B005; **f** B006; **g** B007 and **h** B008 before and after HBV-TCR-T-cell infusion. The numbers of HBV-TCR -T cells are indicated in black, HBsAg and HBV DNA levels are expressed in red and blue, respectively. The blue horizontal lines in **b**, **e**, **f**, **g** and h meant that the levels of HBV DNA levels were lower the limit of detectable levels

Having evaluated the efficacy aspects of the treatment, we next assessed whether the adoptive transfer of the short-lived HBV-TCR-T cells have an on-target effect. By comparing the serum HBsAg levels before and after the last HBV-TCR-T cell infusion, we observed either a decline or stabilization of serum HBsAg in 7 out of 8 patients (Fig. 5a–h). Simultaneously, the circulating HBV DNA loads were reduced or stable at undetectable levels in all 8 patients after TCR-T-cell infusions (Fig. 5a–h). Collectively, these results suggest that the treatment could indeed target HBV-expressing cells.

## Discussion

Previous studies reported that HBV-TCR-T-cell immunotherapy in HBV-HCC patients after liver transplantation with disseminated HCC metastasis in lung, bones and neck or with HCC recurrence had no signs of acute toxicities and only had a largely unremarkable alteration of ALT levels. However, the safety of HBV-TCR-T-cell immunotherapy had not been evaluated in HBV-HCC patients who had not performed liver transplantation. Herein we conducted a dose escalation, phase I trial using short-lived HBV-TCR-T cells in 8 advanced HBV-HCC patients and found that HBV-TCR-T-cell infusions were well-tolerated with one patient (12.5%) experiencing Grade 3 increase of liver enzymes and one patient experiencing Grade 1 AEs with mild elevation of liver enzymes during the first treatment cycle. The AEs resolved without intervention with glucocorticoid and immunosuppressive agents. Compared to CAR-Glypican-3 T-Cell therapy for advanced HCC, the incidence and severity of AEs after HBV-TCR-T-cell immunotherapy in our study was lower and less frequent, and had no neurotoxicity, cytokine release syndrome, or WBC, lymphocyte and platelet count reduction. Comparing to autologous CIK cells for HCC, the rates of AEs were 62%, and the rates of SAEs were 6%. The lower rates of SAEs in CIK therapy than that of HBV-TCR-T-cell immunotherapy may be due to the lower tumor load of patients who had undergone curative treatment. Overall, these data suggest that HBV-TCR-T-cell immunotherapy in advanced HBV-HCC patients without prior liver transplantation was safe and tolerable.

Consistent with the previous study in HBV-HCC patients after liver transplantation, we observed therapeutic efficacy of HBV-TCR-T-cell immunotherapy in the recruited advanced HBV-HCC patients. Three patients showed radiological responses with a shrinkage of tumor, especially in B001 whose tumor size decreased from 13.9 × 6.9 cm to 8.6 × 5.8 cm after four cycles of HBV-TCR-T-cell therapy. This anti-tumor efficacy was likely due to the on-target effects of the adoptively transferred HBV-TCR-T cells as serum HBsAg levels declined in all but one patient after the last HBV-TCR-T cell infusion. Additionally, the median OS duration and the median of time-to-progression in patients were 33.1 and 6.18 months, respectively. Compared to the median OS of advanced patients in our study, the median OS achieved after CAR-Glypican-3 T-Cell therapy (10.0 months) was lower.

There were some limitations in our study. For example, it was very difficult to obtain liver tumor tissues from the enrolled patients due to potential risks that included promoting tumor metastasis and/or bleeding. In addition, there was a small number of enrolled patients. Future studies are warranted to confirm the safety and efficacy of such short-lived HBV-TCR-T cells in a larger cohort of advanced HBV-HCC patients.

In conclusion, adoptive T cell therapy using autologous short-lived HBV-TCR-T cells is a technically feasible and safe treatment with tolerable toxicity and with potential therapeutic efficacy in advanced HBV-HCC patients. These data accumulated thus far support the continued development of this approach as an alternative treatment for HCC.

## Abbreviations

ALT: Alanine aminotransferase
HBV-TCR-T cells: HBV-specific TCR-redirected T cells
BCLC: Barcelona clinic liver cancer stage
CIK: Cytokine-induced killer cells
ECOG: Eastern cooperative oncology group
HBsAg: Hepatitis B surface antigen
HBV: Hepatitis B virus
HCC: Hepatocellular carcinoma
ICB: Immune checkpoint blockade
OS: Overall survival
TTP: Time-to-progression
TACE: Transhepatic arterial chemotherapy and embolization
PBMCs: Peripheral blood mononuclear cells

## Declarations

### Human and animal research

This article does not contain any studies with animals performed by any of the authors.
